# The Fundamental Structure and the Reproduction of Spiral Wave in a Two-Dimensional Excitable Lattice

**DOI:** 10.1371/journal.pone.0149842

**Published:** 2016-02-22

**Authors:** Yu Qian, Zhaoyang Zhang

**Affiliations:** 1 Nonlinear Research Institute, Baoji University of Arts and Sciences, Baoji, Shaanxi, China; 2 Department of Physics, Faculty of Science, Ningbo University, Ningbo, Zhejiang, China; Lanzhou University of Technology, CHINA

## Abstract

In this paper we have systematically investigated the fundamental structure and the reproduction of spiral wave in a two-dimensional excitable lattice. A periodically rotating spiral wave is introduced as the model to reproduce spiral wave artificially. Interestingly, by using the dominant phase-advanced driving analysis method, the fundamental structure containing the loop structure and the wave propagation paths has been revealed, which can expose the periodically rotating orbit of spiral tip and the charity of spiral wave clearly. Furthermore, the fundamental structure is utilized as the core for artificial spiral wave. Additionally, the appropriate parameter region, in which the artificial spiral wave can be reproduced, is studied. Finally, we discuss the robustness of artificial spiral wave to defects.

## Introduction

A two-dimensional (2D) regular lattice with local cells persisting excitable dynamics is called an excitable medium [1, 2]. Spatiotemporal pattern formation in excitable media is one of the most important issues in nonlinear science, and has attracted great attention during the last thirty years due to its relevance to various important systems, such as cardiac tissues and neural networks for typical examples [3–8]. Although single excitable cell is not oscillatory, self-organized oscillatory spatiotemporal patterns, however, are extremely important in these systems. Spiral wave is one of the most important and typical spatiotemporal patterns in excitable media and can self-sustain in autonomous systems. Spiral wave and spiral wave instability in cardiac tissues are associated with pathological types of wave dynamics [3]. Therefore, many effective methods have been proposed to control spiral wave and spiral wave instability in cardiac tissues [9–20]. For example, Garfinkel et al. studied the prevention of ventricular fibrillation by flattening cardiac restitution [11]. Zhang et al. investigated the suppression of spiral waves and spatiotemporal chaos by generating target waves in excitable media [13]. Allexandre et al. presented an ion-channel-based approach to prevent alternans-induced spiral wave breakup in cardiac tissue [15]. Lou et al. studied the control of turbulence in heterogeneous excitable media [18].

Recently, people have reported the observation of spiral wave in the mammalian neocortex. Experimentally, Huang et al. observed spiral waves in mammalian cortex [21, 22]. Schiff et al. studied dynamical evolution of spiral waves in mammalian middle cortex [23]. Accordingly, many prominent works have been taken on the spatiotemporal dynamics of spiral waves in neuronal networks. Lots of interesting phenomena have been discovered in recent years [24–31]. For example, Perc investigated the effects of small-world connectivity on noise-induced temporal and spatial order in neural media [24]. Wang et al. studied time delay enhanced coherence of spiral waves in noisy Hodgkin-Huxley neuronal networks [25]. Moreover, a series of contributions in this field was achieved by Ma et al. [26–31]. They reported the formation, the death, the breakup and the transition of spiral waves in neuronal networks. These excellent achievements can help us to explore the significant roles of spiral waves in brain systems.

As the concepts of “small-world” [32] and “scale-free” [33] had been proposed by Watts and Barabási in the last century, remarkable advances have been achieved in the field of complex network in recent years [34, 35]. Problems of spatiotemporal pattern formation in excitable complex networks have become one of the central topics under investigation. Self-sustained oscillation is one of the most important issues in this field. Since, oscillations in neural networks and brain systems are related to some specific and important physiological functions, such as visual perception [36], olfaction [37], cognitive processes [38], sleep and arousal [39] and so on. Theoretically, several significant contributions related to the phenomena and mechanisms of self-sustained oscillations in excitable complex networks have been achieved in recent decades [40–51]. For example, Roxin et al. investigated the self-sustained activity in a small-world network of excitable neurons [40]. The emergence of self-sustained patterns in small-world excitable media was reported by Sinha et al. [45]. In our previous works [47, 49], the dominant phase-advanced driving (DPAD) analysis method was proposed to investigate the periodically self-sustained oscillations in excitable complex networks. By using the DPAD method, the fundamental structures containing the oscillation sources (the one-dimensional (1D) Winfree-loops [52]) and the wave propagation paths, which can self-organize in the networks, have been revealed to maintain the oscillations. And the fundamental structure can instruct us to suppress or to reproduce the oscillation effectively. As stated above, spiral wave can self-sustain in autonomous excitable media. Consequently, the following questions arises: Whether similar fundamental structure can also emerge in spiral wave? If yes, whether the fundamental structure can instruct us to suppress or to reproduce spiral wave effectively? All of these are the tasks we try to explore in present paper.

## Mathematical Model and Setup

In this paper, a 2D regular excitable lattice containing 50 × 50 nodes is considered. The Bär-Eiswirth [53] model is adopted for local dynamics. The evolution of the studied 2D excitable lattice dynamics is described by the following equations:

$$\frac{\text{d}u_{i,j}}{\text{d}t}=-\frac{1}{\varepsilon }u_{i,j}\left(u_{i,j}-1\right)\left(u_{i,j}-\frac{v_{i,j}+b}{a}\right)+D\left(u_{i-1,j}+u_{i+1,j}+u_{i,j-1}+u_{i,j+1}-4u_{i,j}\right),$$ (1)

$$\frac{\text{d}v_{i,j}}{\text{d}t}=f\left(u_{i,j}\right)-v_{i,j},$$ (2)

where *i*, *j* = 1, 2, …, 50. The function *f*(*u*_*i*, *j*_) takes the form: *f*(*u*_*i*, *j*_) = 0 for $u_{i,j}<\frac{1}{3}$; *f*(*u*_*i*, *j*_) = 1 − 6.75*u*_*i*, *j*_(*u*_*i*, *j*_ − 1)^2^ for $\frac{1}{3}\leq u_{i,j}\leq 1$; and *f*(*u*_*i*, *j*_) = 1 for *u*_*i*, *j*_ > 1. In Eqs (1) and (2), variables *u* and *v* describe the activator and the inhibitor, respectively. The small relaxation parameter *ϵ* represents the time ratio between activator *u* and inhibitor *v*. The dimensionless parameters *a* and *b* denote the activator kinetics with *b* effectively controlling the excitation threshold. The system parameters are kept throughout this paper as *a* = 0.84, *b* = 0.07 and *ϵ* = 0.04. Consequently, each node in the lattice can follow a typically excitable dynamics. Here *D* is the diffusion coefficient of activator *u*. The above dynamical equations are integrated by the forward Euler integration scheme with time step Δ*t* = 0.03 and no-flux boundary condition is used.

As we will use the DPAD method to analyze the fundamental structure of spiral wave in the next section, the main idea of the DPAD method is briefly interpreted here. The dominant phase-advanced driving method was proposed to analyze the fundamental structures of periodically self-sustained oscillations in excitable complex networks. The basic idea of the DPAD method is as follows. It is well known that the single excitable node is not self-oscillatory, it can oscillate if and only if it is driven by one or few oscillatory neighbouring interactions with advanced phases. Among all these phase-advanced interactions, the interaction providing the most contribution to exciting the given node, is defined as the dominant phase-advanced driving. Based on this idea, the corresponding DPAD relationship for each excitable node can be identified. Then the original excitable complex network can be reduced to structurally simple and instructive subnetwork of unidirectional DPAD paths. Consequently, the fundamental structures of self-sustained oscillations, which contains the oscillation sources and the wave propagation paths, can be revealed by DPAD patterns explicitly.

## Results

### The fundamental structure of periodically rotating spiral wave

**Fig 1:**
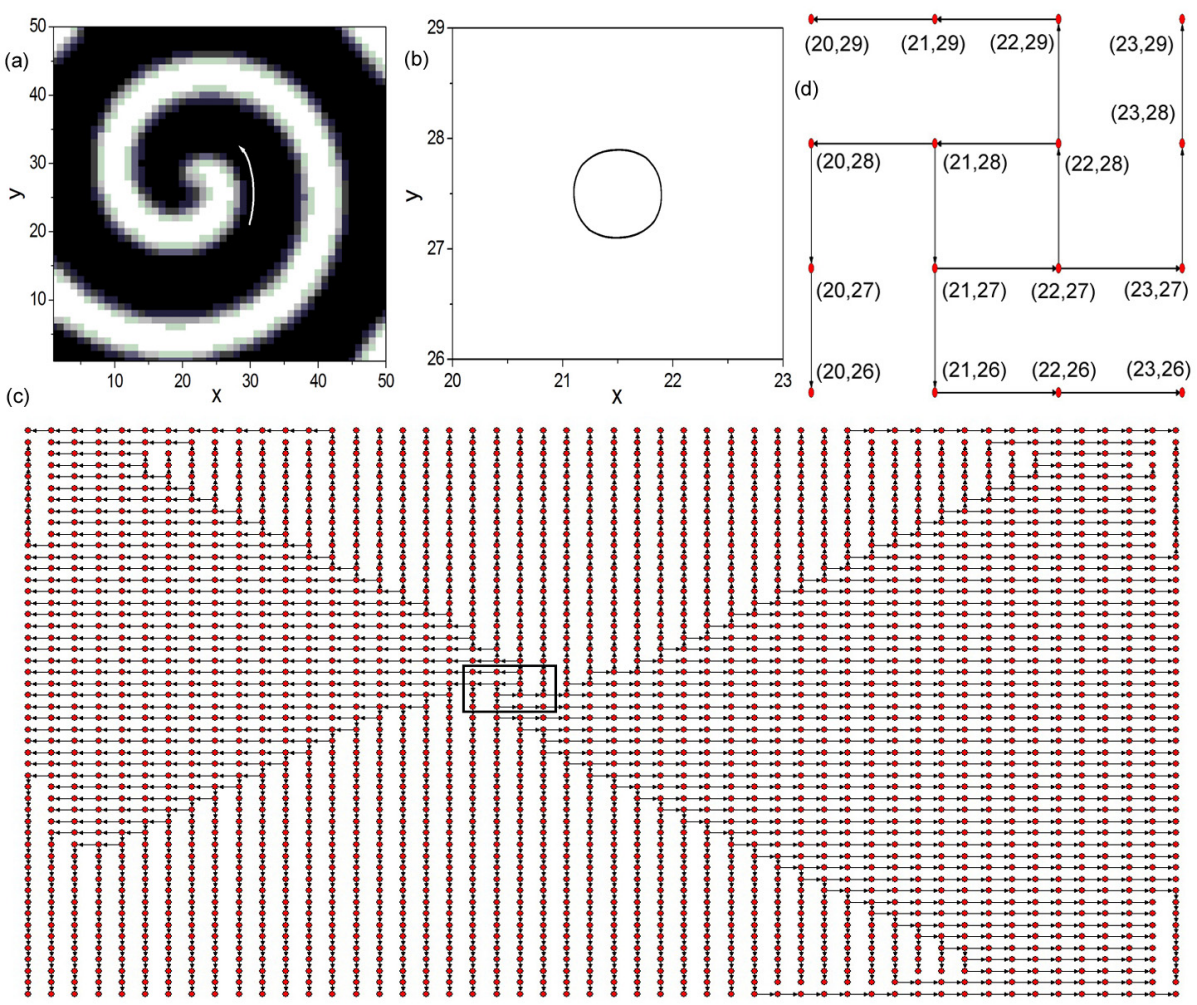
The fundamental structure of periodically rotating spiral wave. (a) A periodically rotating spiral wave is introduced in a two-dimensional (2D) regular excitable lattice containing 50 × 50 nodes at diffusion coefficient *D* = 4.0. Other system parameters are kept throughout this paper as *a* = 0.84, *b* = 0.07 and *ϵ* = 0.04. The figure is plotted in greyscale from black (lowest value at 0.0) to white (highest value at 1.0). And this greyscale will be used throughout this paper. The white arrowed line indicates the rotation direction of the spiral wave. (b) The tip trajectory of spiral wave (a). In present paper, spiral tip is defined to be the intersection of the two contours *u* = 0.50 and *v* = 0.35. Here *v* = 0.35 is calculated according to the formula: *g*(*u* = 0.50, *v*) = 0, where $g\left(u,v\right)=-\frac{1}{\varepsilon }u\left(u-1\right)\left(u-\frac{v+b}{a}\right)$ is the local dynamics of variable *u*. (c) The dominant phase-advanced driving (DPAD) pattern corresponding to the spiral wave (a). The red dots represent the nodes in the 2D regular excitable lattice, and the black arrowed lines denote the wave propagation paths. (d) The local amplified DPAD pattern of the black rectangular area in (c). The index, such as the (21, 28), represents the node position in the 2D regular excitable lattice with *x* = 21 and *y* = 28. The fundamental structure of periodically rotating spiral wave, which contains the loop structure and the wave propagation paths, is revealed by the DPAD analysis method clearly.

In this part, we firstly analyze the fundamental structure of periodically rotating spiral wave. A spiral wave is introduced in a 2D regular excitable lattice containing 50 × 50 nodes at diffusion coefficient *D* = 4.0, as shown in Fig 1(a). The white arrowed line denotes the rotation direction of the spiral pattern. It is shown that the spiral wave introduced in Fig 1(a) rotates in the counterclockwise direction. Fig 1(b) exhibits the tip trajectory of the spiral wave. In present paper, spiral tip is defined to be the intersection of the two contours *u* = 0.50 and *v* = 0.35. Here *v* = 0.35 is calculated according to the formula: *g*(*u* = 0.50, *v*) = 0, where $g\left(u,v\right)=-\frac{1}{\varepsilon }u\left(u-1\right)\left(u-\frac{v+b}{a}\right)$ is the local dynamics of variable *u*. It is shown that the tip trajectory is simply a circle, which indicates the spiral wave introduced in Fig 1(a) is a periodically rotating spiral wave.

Now we try to use the DPAD method to analyze the fundamental structure of the periodically rotating spiral wave. Fig 1(c) reveals the DPAD pattern corresponding to the spiral wave of Fig 1(a). The red dots represent the nodes in the 2D regular excitable lattice, and the black arrowed lines denote the wave propagation paths. Fig 1(d) displays the local amplified DPAD pattern of the black rectangular area in Fig 1(c). It is shown that the excitable wave propagates along the pathway (21, 28) → (21, 27) → (22, 27) → (22, 28) → (21, 28) to form an unidirectional loop structure. Here index (21, 28) represents the node position in the 2D regular excitable lattice with *x* = 21 and *y* = 28. By using the DPAD analysis method, a loop structure is revealed for spiral wave.

**Fig 2:**
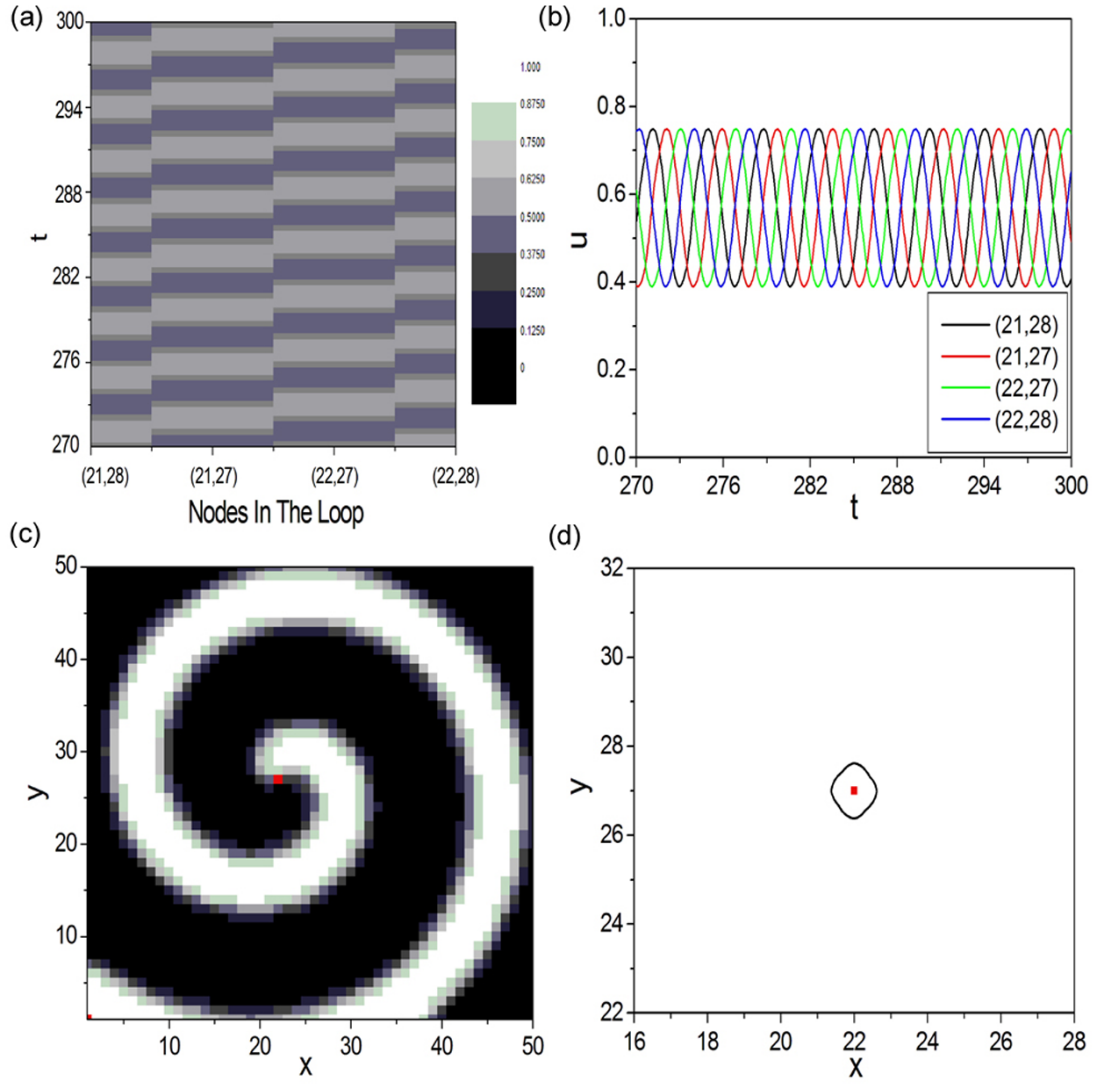
To check whether the loop structure discovered in spiral wave is the same as the source loop in oscillatory excitable complex network. (a) (b) The space-time plot ((a)) and time series ((b)) of variable *u* of nodes in the loop structure of spiral wave. These nodes are ordered according to the sequence in the loop structure. (c) (d) The spiral pattern ((c)) and the tip trajectory ((d)) obtained as the loop structure of spiral wave is destroyed. In this paper we break the loop structure by discarding the node in the loop. The node (22, 27) in the loop structure is discarded, as shown by the red node in (c) and (d).

As we know that, in oscillatory excitable complex network, excitable wave can propagate along the loop structure to form 1D Winfree-loop serving as the oscillation source to maintain the oscillation. Hence, the loop structure revealed in oscillatory excitable complex network is defined as the source loop. And the self-sustained oscillation will damp if the source loop is destroyed. Now we would ask whether the loop structure discovered in spiral wave (shown by Fig 1(d)) is the same as the source loop in oscillatory excitable complex network? To answer the above question, the spatiotemporal evolution pattern and the time series of nodes in the loop structure of spiral wave are firstly investigated, which are displayed in Fig 2(a) and 2(b), respectively. These nodes are ordered according to the sequence in the loop structure. Although wave propagation pattern is obtained (shown by Fig 2(a)), the nodes in the loop structure do not oscillate from the rest state (shown by Fig 2(b)). It is well known that the nodes in the 1D Winfree-loop should oscillate from the rest state to the excited state. So we consider the loop structure discovered in spiral wave is not the well-known 1D Winfree-loop. Now we will try to destroy the loop structure to check whether it exists as the oscillation source for spiral wave. In this paper we break the loop structure by discarding the node in the loop. Fig 2(c) and 2(d) show the spiral pattern (shown by Fig 2(c)) and the tip trajectory (shown by Fig 2(d)) obtained as the loop structure shown in Fig 1(d) is destroyed. Node (22, 27) in the loop structure is discarded, as shown by the red node in Fig 2(c) and 2(d). It is shown that the spiral wave survives and rotates around the discarded node as the loop structure is destroyed, which reveals that the loop structure discovered by the DPAD analysis method is not the oscillation source of spiral wave. It is entirely different from the source loop revealed in oscillatory excitable complex network, which exists as the oscillation source for self-sustained oscillation.

Although the loop structure revealed by the DPAD analysis method is not the oscillation source of spiral wave, other characteristics of spiral wave can be indicated by the DPAD pattern. By comparing Fig 1(b) and 1(d) we can find that the loop structure locates in the spiral tip region. The node in the loop structure is the actual path, along which the spiral tip passes through in the 2D lattice. So the periodically rotating orbit of spiral tip can be exposed by the loop structure clearly. Meanwhile, the excitable wave on the loop structure propagates in the counterclockwise direction, which is the same as the rotation direction of the spiral wave (counterclockwise direction, denoted by the white arrowed line in Fig 1(a)). Hence the charity of spiral wave can be indicated by the unidirectional loop structure explicitly. Additionally, the unidirectional wave propagation path outside the loop structure shows the way, in which the spiral wave propagates from the spiral tip to the boundary. Based on the above discussions, we can conclude that the fundamental structure of periodically rotating spiral wave, which contains the loop structure and the wave propagation paths, is revealed by the DPAD analysis method. Furthermore, it can indicate the periodically rotating orbit of spiral tip and the charity of spiral wave clearly. More importantly, we will use the fundamental structure as the core to reproduce spiral wave artificially.

### Reproduction of artificial spiral wave

To reproduce the artificial spiral wave successfully, the oscillation source and the network structure supporting the wave propagation of artificial spiral wave are two necessary conditions. Although the loop structure revealed in Fig 1(c) is not the oscillation source of spiral wave, we can use it as the source loop for artificial spiral wave (similar to the 1D Winfree-loop supporting the self-sustained oscillation in excitable complex network). And the wave propagation path outside the loop structure can be used to construct unidirectional complex network for supporting the wave propagation of artificial spiral wave. Based on the above understandings, now we can try to use the fundamental structure of periodically rotating spiral wave to reproduce spiral wave artificially. Firstly, we use the DPAD pattern shown in Fig 1(c) to construct unidirectional excitable complex network to form the skeleton structure for artificial spiral wave. Secondly, we try to give excitations to the nodes in the loop structure to form 1D Winfree-loop to support the artificial spiral wave. However, no 1D Winfree-loop forms. All excitations damp to the rest state quickly. It indicates that the 1D Winfree-loop can not self-organize on too small excitable loop (the loop structure shown in Fig 1(c) only contains 4 excitable nodes). This is induced by the existence of the refractory period of excitable dynamics. The excitable loop must be sufficiently large to maintain the recurrent excitation to form 1D Winfree-loop. Therefore, to find the minimal 1D Winfree-loop length under current parameters will be of great importance.

**Fig 3:**
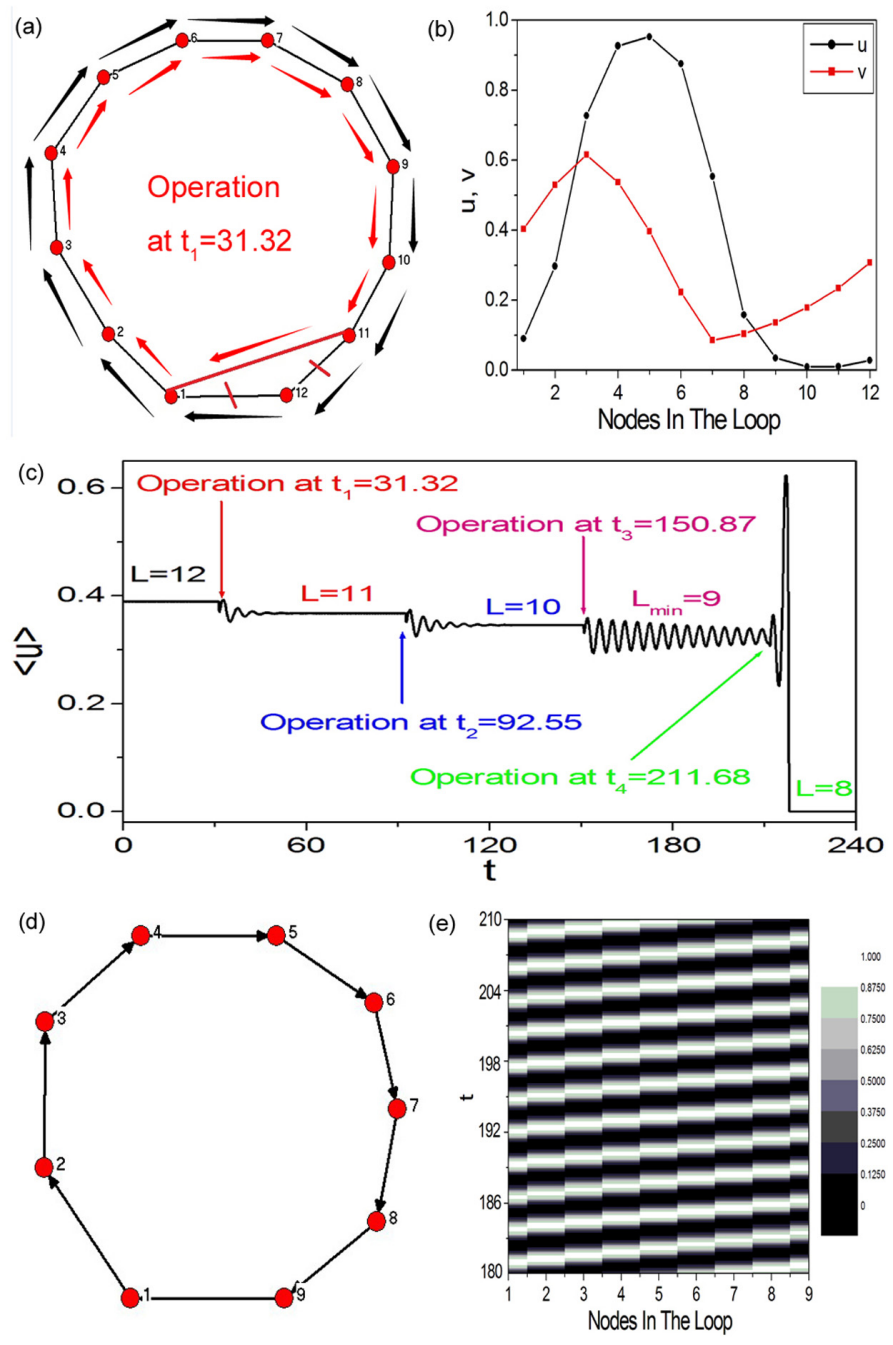
The schematic diagram of the way we calculate the minimal one-dimensional (1D) Winfree-loop length *L*_*min*_. (a) An artificial 1D periodic excitable ring containing 12 nodes is constructed. (b) The wave form on a 1D periodic excitable ring containing 12 nodes, which will be used as the initial condition for (a). When the wave form shown in (b) is applied, unidirectional wave propagation is formed along the pathway 1 → 2 → 3 → 4 → 5 → 6 → 7 → 8 → 9 → 10 → 11 → 12 → 1 to form 1D Winfree-loop in (a) (indicated by the outside black arrowed lines). As the peak of the excitable wave passes through node 1, the following operation is executed: Node 12 in (a) is discarded from the 1D excitable ring (i.e., discarding the connections between node 12 and other nodes, denoted by the two red short lines). Simultaneously, a connection between nodes 1 and 11 is added (denoted by the red long line). The operation is executed at *t* = 31.32. Then, a new shorter 1D Winfree-loop composed by 1 → 2 → 3 → 4 → 5 → 6 → 7 → 8 → 9 → 10 → 11 → 1 self-organizes to support the oscillation (indicated by the inside red arrowed lines). (c) Trajectory of $<u\left(t\right)>=\frac{1}{N}\sum_{i=1}^{N=12}u_{i}\left(t\right)$ as the above operation is executed successively at *t*_1_ = 31.32, *t*_2_ = 92.55, *t*_3_ = 150.87 and *t*_4_ = 211.68, respectively. As the fourth operation is executed at *t*_4_ = 211.68, < *u*(*t*) > damps to zero quickly, which indicates that the minimal 1D Winfree-loop length supporting self-sustained oscillation at diffusion coefficient *D* = 4.0 is found to be 9 (i.e., *L*_*min*_ = 9 at *D* = 4.0). (d) (e) The DPAD pattern ((d)) and the space-time plot ((e)) corresponding to the minimal 1D Winfree-loop containing 9 excitable nodes.

Fig 3(a) exhibits the schematic diagram of the way we calculate the minimal 1D Winfree-loop length *L*_*min*_. An artificial 1D periodic excitable ring containing 12 nodes is constructed. Fig 3(b) shows the wave form on a 1D periodic excitable ring containing 12 nodes, which will be used as the initial condition for Fig 3(a). When the wave form shown in Fig 3(b) is applied, unidirectional wave propagation is formed along the pathway 1 → 2 → 3 → 4 → 5 → 6 → 7 → 8 → 9 → 10 → 11 → 12 → 1 to form 1D Winfree-loop in Fig 3(a) (indicated by the outside black arrowed lines). As the peak of the excitable wave passes through node 1, the following operation is executed: Node 12 in Fig 3(a) is discarded from the 1D excitable ring (i.e., discarding the connections between node 12 and other nodes, denoted by the two red short lines). Simultaneously, a connection between nodes 1 and 11 is added (denoted by the red long line). The operation is executed at *t* = 31.32. Then, a new shorter 1D Winfree-loop composed by 1 → 2 → 3 → 4 → 5 → 6 → 7 → 8 → 9 → 10 → 11 → 1 self-organizes to support the oscillation (indicated by the inside red arrowed lines). Fig 3(c) shows the trajectory of $<u\left(t\right)>=\frac{1}{N}\sum_{i=1}^{N=12}u_{i}\left(t\right)$ as the above operation is executed successively at *t*_1_ = 31.32, *t*_2_ = 92.55, *t*_3_ = 150.87 and *t*_4_ = 211.68, respectively. As the fourth operation is executed at *t*_4_ = 211.68, <*u*(*t*)> damps to zero quickly. It indicates that the minimal 1D Winfree-loop length supporting the oscillation at diffusion coefficient *D* = 4.0 is found to be 9 (i.e., *L*_*min*_ = 9 at *D* = 4.0). Fig 3(d) and 3(e) display the DPAD pattern and the space-time plot corresponding to the minimal 1D Winfree-loop containing 9 excitable nodes, respectively. From the results shown in Fig 3, we can conclude that the loop structure shown in Fig 1(c) containing 4 excitable nodes is too small to form 1D Winfree-loop supporting the artificial spiral wave under current parameters.

**Fig 4:**
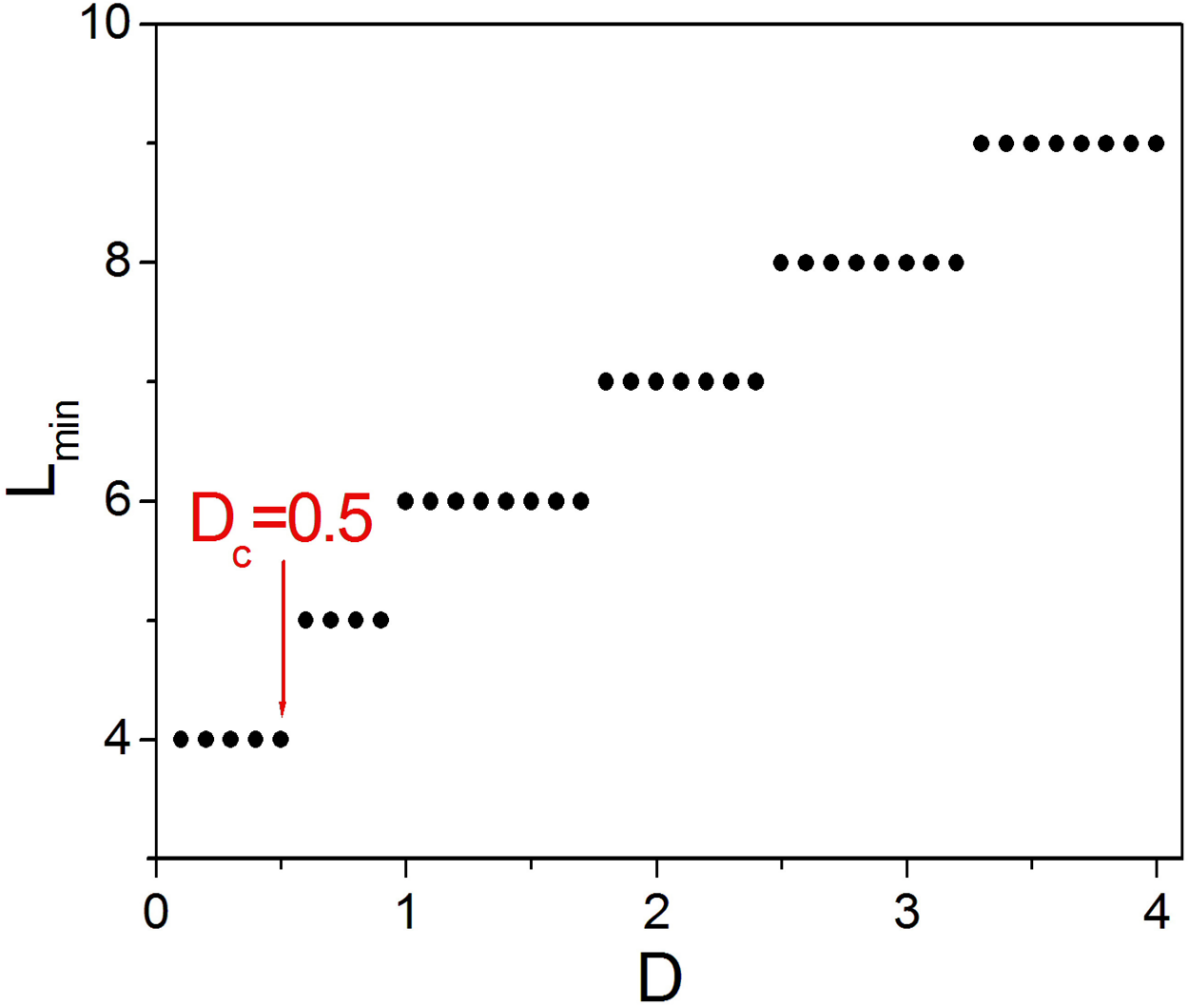
The investigation of the appropriate parameter region, in which the artificial spiral wave can be reproduced. Dependence of the minimal 1D Winfree-loop length *L*_*min*_ on diffusion coefficient *D*. It is shown *L*_*min*_ = 4 for *D* ≤ 0.5, which indicates the appropriate parameter region for reproducing spiral wave artificially.

To form 1D Winfree-loop on the loop structure containing 4 excitable nodes, the dependence of the minimal 1D Winfree-loop length *L*_*min*_ on system parameter needs to be investigated. Fig 4 shows the relationship between the minimal 1D Winfree-loop length *L*_*min*_ and diffusion coefficient *D*. It is shown *L*_*min*_ = 4 for *D* ≤ 0.5, which indicates the appropriate parameter region for reproducing spiral wave artificially.

**Fig 5:**
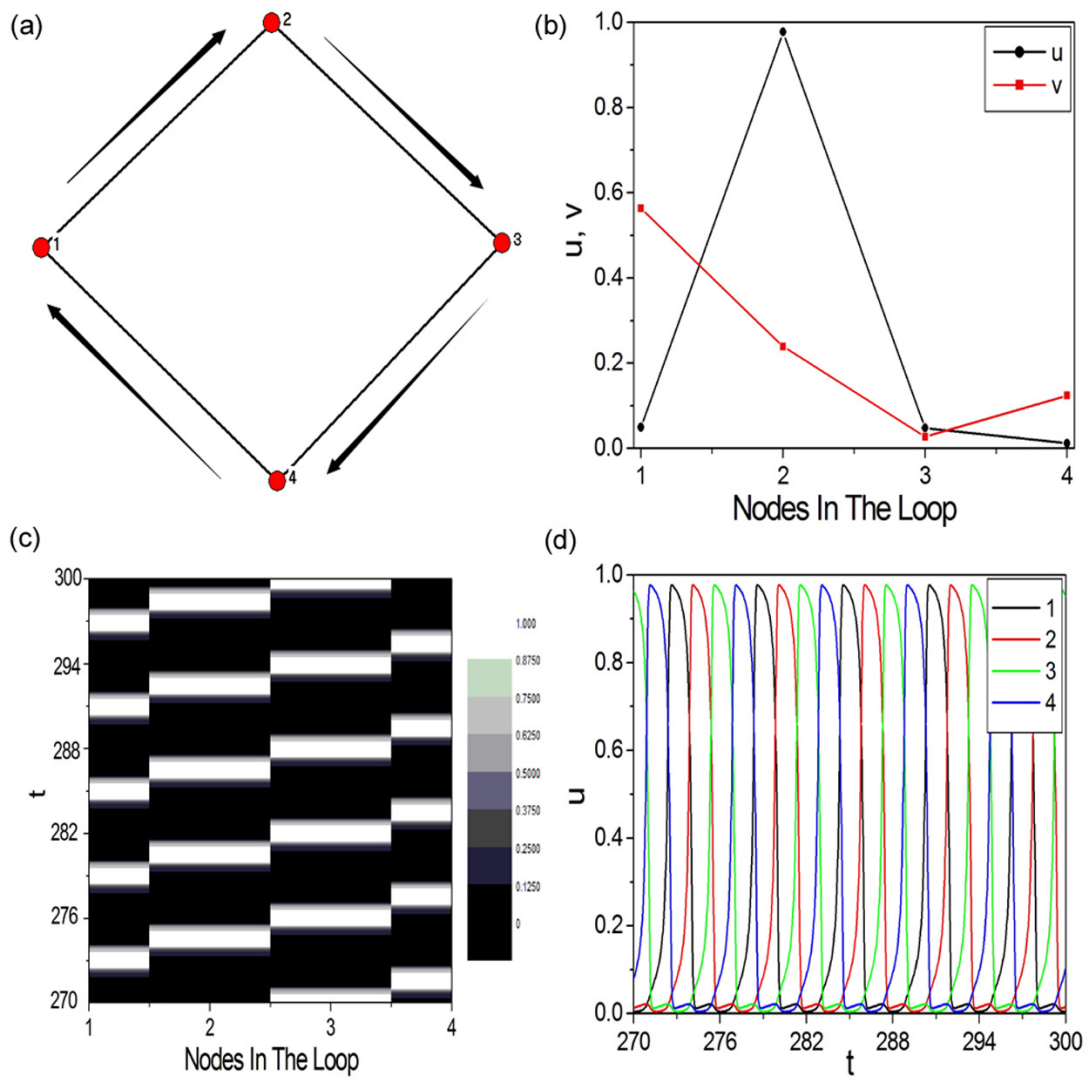
The minimal 1D Winfree-loop at diffusion coefficient *D* = 0.2. An artificial 1D periodic excitable ring containing 4 nodes is constructed at diffusion coefficient *D* = 0.2. (b) The wave form on a 1D periodic excitable ring containing 4 nodes, which will be used as the initial condition for (a). When the wave form shown in (b) is applied, unidirectional wave propagation is formed along the pathway 1 → 2 → 3 → 4 → 1 to form 1D Winfree-loop in (a) (indicated by the outside black arrowed lines). (c) (d) The space-time plot ((c)) and the time series ((d)) of variable *u* of nodes in the 1D Winfree-loop containing 4 excitable nodes.

To further check the above results, an artificial 1D periodic excitable ring containing 4 nodes is constructed at diffusion coefficient *D* = 0.2, which is shown in Fig 5(a). Fig 5(b) displays the wave form on a 1D periodic excitable ring containing 4 nodes, which will be used as the initial condition for Fig 5(a). When the wave form shown in Fig 5(b) is applied, the excitable wave can propagate along the unidirectional pathway 1 → 2 → 3 → 4 → 1 to form 1D Winfree-loop containing 4 excitable nodes (indicated by the outside black arrowed lines in Fig 5(a)). Fig 5(c) and 5(d) display the space-time plot and the time series of nodes in the 1D Winfree-loop containing 4 excitable nodes, respectively. Perfect wave propagation pattern is obtained (shown by Fig 5(c)), and the nodes in the loop structure oscillate from the rest state to the excited state (shown by Fig 5(d)). Up to now, the 1D Winfree-loop containing 4 excitable nodes has been obtained, which can be used as the oscillation source to maintain the artificial spiral wave.

**Fig 6:**
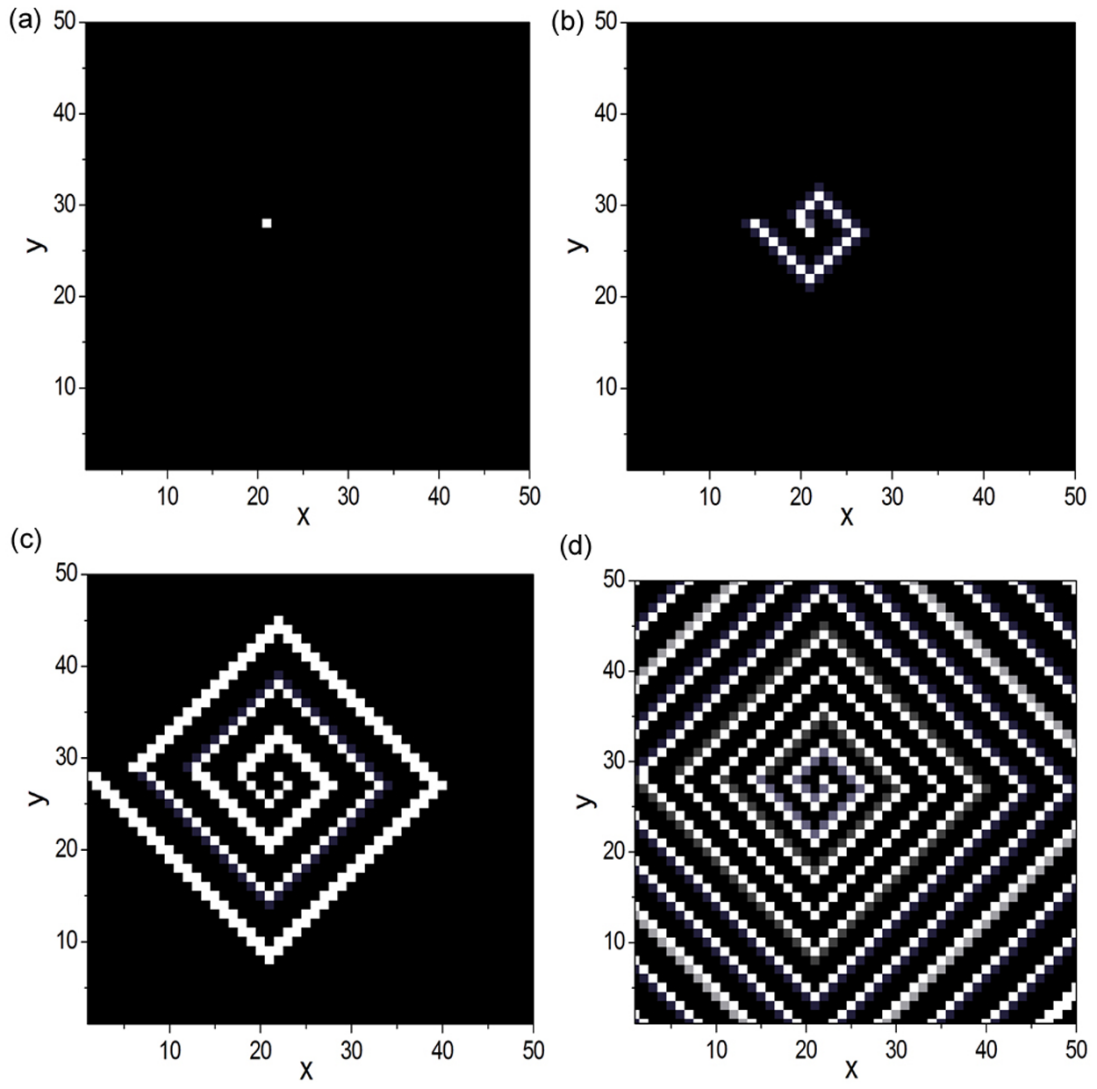
Reproduction of artificial spiral wave. The evolvement of the artificial spiral wave at diffusion coefficient *D* = 0.2. The snapshots are obtained at *t* = 0 (a), 7.5 (b), 22.5 (c) and 75 (d), respectively. The DPAD pattern shown in Fig 1(c) is used to construct unidirectional excitable complex network to form the skeleton structure for artificial spiral wave. The initial excitation is stimulated on node (21, 28), which is located in the loop structure. The artificial spiral wave has been reproduced successfully in numerical simulation.

Based on the above discussions, now we can reproduce spiral wave under appropriate system parameters. Here we choose diffusion coefficient *D* = 0.2. We firstly use the DPAD pattern shown in Fig 1(c) to construct unidirectional excitable complex network to form the skeleton structure for artificial spiral wave. Then, we give initial excitation to the node in the loop structure (the initial excitation is stimulated on node (21, 28)). Fig 6(a)–6(d) display the evolvement of the artificial spiral wave at diffusion coefficient *D* = 0.2. The snapshots are obtained at *t* = 0 (shown by Fig 6(a)), 7.5 (shown by Fig 6(b)), 22.5 (shown by Fig 6(c)) and 75 (shown by Fig 6(d)), respectively. It is shown that the initial excitation can propagate along the loop structure to form 1D Winfree-loop serving as the oscillation source. And the excitable wave can propagate from the source loop to the boundary along the skeleton structure continuously as time evolves. Till now, spiral wave has been reproduced artificially in numerical simulation.

### Robustness of artificial spiral wave to defects

**Fig 7:**
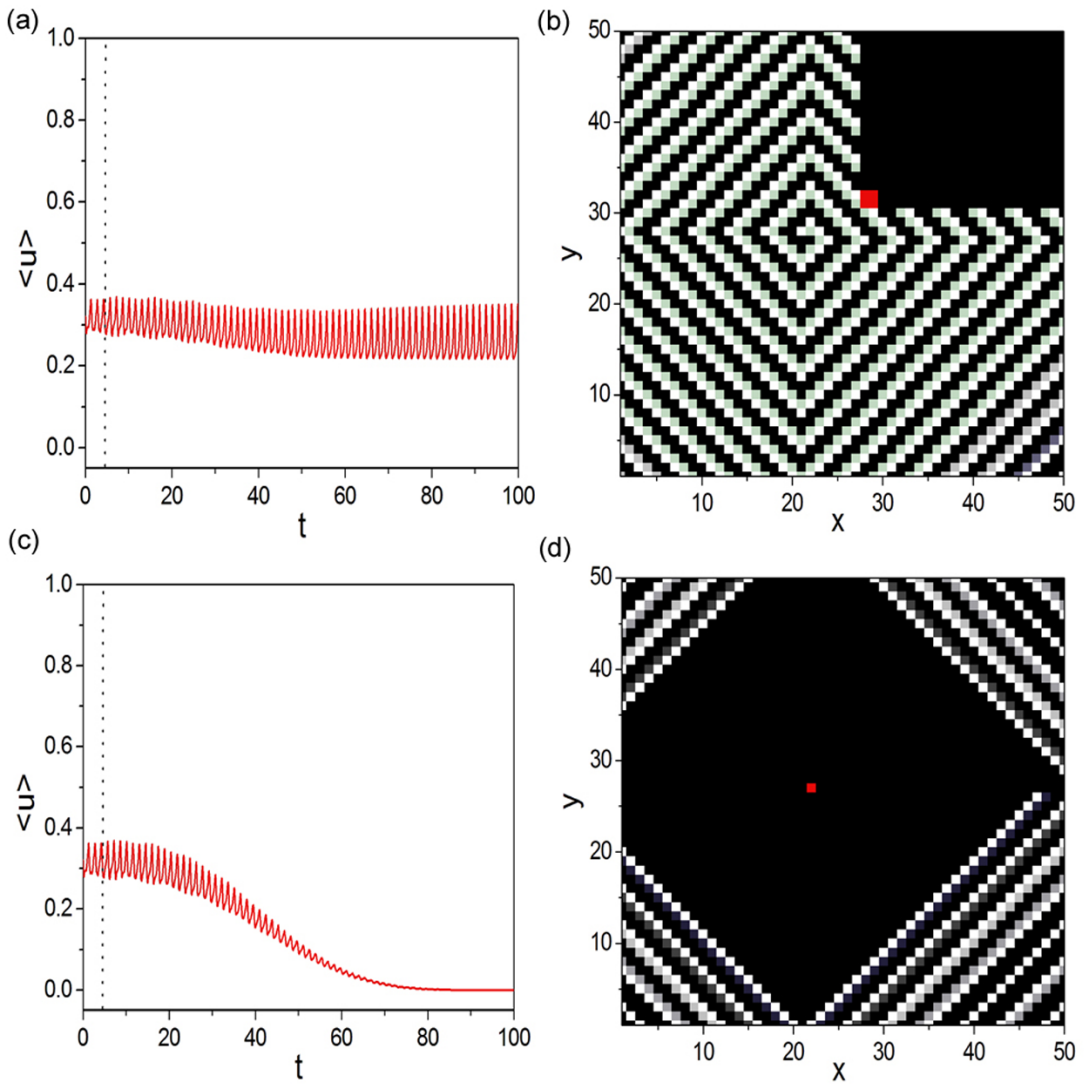
Robustness of artificial spiral wave to defects. (a) (b) Trajectories of $<u\left(t\right)>=\frac{1}{N^{2}}\sum_{i=1}^{N^{2}\left(N=50\right)}u_{i}\left(t\right)$ ((a)) and the spatiotemporal pattern ((b)) obtained as the 2 × 2 square defect (denoted by the red region in (b)) outside the 1D Winfree-loop is introduced at *t* = 4.5 (indicated by the dot line in (a)). The spatiotemporal pattern shown in Fig 6(d) is used as the initial condition for artificial spiral wave. In present paper, defects are introduced by discarding all connections between nodes in defect region and outside defect region. Artificial spiral wave is robust to the defect outside the oscillation source. (c) (d) The same as (a) and (b) when the defect is introduced on the 1D Winfree-loop. As the defect on the source loop is introduced, < *u*(*t*) > damps to zero gradually. The spatiotemporal pattern shown in (d) is obtained at *t* = 45, where the artificial spiral wave approaches homogeneous rest state. The artificial spiral wave degenerates as the oscillation source is destroyed.

In this part, we discuss the robustness of artificial spiral wave to defects. The spatiotemporal pattern shown in Fig 6(d) is used as the initial condition for artificial spiral wave. The defects will be introduced outside the 1D Winfree-loop and on the 1D Winfree-loop, respectively. In present paper, defects are introduced by discarding all connections between nodes in defect region and outside defect region. Fig 7(a) and 7(b) display the trajectories of $<u\left(t\right)>=\frac{1}{N^{2}}\sum_{i=1}^{N^{2}\left(N=50\right)}u_{i}\left(t\right)$ (shown by Fig 7(a)) and the spatiotemporal pattern (shown by Fig 7(b)) obtained as the 2 × 2 square defect outside the 1D Winfree-loop is introduced. The defect is located at the red region in Fig 7(b) and is introduced at *t* = 4.5 (indicated by the dot line in Fig 7(a)). It is shown that < *u*(*t*) > can still maintain oscillation as the defect is introduced. It indicates that the artificial spiral wave is robust to the defect outside the oscillation source. Now we study the robustness of artificial spiral wave to defect on the 1D Winfree-loop. Fig 7(c) and 7(d) show the results when the defect is introduced on the oscillation source. As the defect is introduced, < *u*(*t*) > damps to zero gradually (shown by Fig 7(c)). The spatiotemporal pattern shown in Fig 7(d) is obtained at *t* = 45, where the artificial spiral wave approaches homogeneous rest state. It is shown that the artificial spiral wave degenerates as the oscillation source is destroyed.

Based on the formation mechanism of artificial spiral wave, we can well explain the results obtained in Fig 7. As the defect is introduced outside the 1D Winfree-loop (shown by the red region in Fig 7(b)), the oscillation source is not influenced. The excitable wave can propagate along the loop structure to form 1D Winfree-loop to main the oscillation of artificial spiral wave. Meanwhile, since the unidirectional excitable complex network is constructed as the skeleton structure for artificial spiral wave, the downstream nodes which are driven by the original red region cells will damp as the defect is introduced. So the spatiotemporal pattern shown in Fig 7(b) can be obtained; As the defect is introduced on the 1D Winfree-loop (shown by the red node in Fig 7(d)), the oscillation source is destroyed. No 1D Winfree-loop can form under this circumstance. Without excitations from oscillation source, outside excitable nodes cease to excite. When the original artificial spiral wave flows out the boundary, < *u*(*t*) > damps to zero gradually (shown by Fig 7(c)) and the system finally evolves into the homogeneous rest state.

## Conclusion

In conclusion, the fundamental structure and the reproduction of spiral wave in a two-dimensional excitable lattice has been systematically investigated in this paper. Firstly, a periodically rotating spiral wave is introduced in the middle of medium and is used as the model to reproduce spiral wave artificially. By using the DPAD analysis method, the fundamental structure containing the loop structure and the wave propagation paths has been revealed. Interestingly, it can expose the periodically rotating orbit of spiral tip and the charity of spiral wave clearly. Furthermore, the fundamental structure is utilized as the core for artificial spiral wave. Then, the appropriate parameter region, in which the artificial spiral wave can be reproduced, is studied. Based on the above investigations, the artificial spiral wave is reproduced successfully in numerical simulation. Finally, we discuss the robustness of artificial spiral wave to defects. It is shown that the artificial spiral wave is robust to the defect outside the oscillation source, and will degenerate as the defect is introduced on the source loop.

As we know that spiral patterns in excitable media are very important issues in a wide variety practical fields, especially in cardiac tissues and neural networks, and may be related to some specific physiological functions, a systematical investigation of the fundamental structure and the reproduction of spiral wave is expected to be useful both for theoretical understandings and practical applications. We do hope our work will be a useful supplement to the previous contributions and will have a useful impact in related fields.
